# Enamel Matrix Derivative (EMD) as an Adjunct to Non-surgical Periodontal Therapy: A Systematic Review

**DOI:** 10.7759/cureus.43530

**Published:** 2023-08-15

**Authors:** Mahmoud Abu-Ta'a, Dina Marzouka

**Affiliations:** 1 Dental Sciences, Arab American University, Ramallah, PSE

**Keywords:** root planing, scaling, regeneration, surgical therapy, non-surgical periodontal therapy, periodontitis, enamel matrix derivative

## Abstract

If left untreated, periodontitis is a chronic, irreversible disease that can contribute to tooth loss. The primary objective of periodontal treatment is to arrest the progression of the disease and restore the supporting structures of the tooth. Scaling and root planing (SRP) is a common non-surgical periodontal therapy (NSPT) used to reduce inflammation, pocket depth, and clinical attachment loss. However, NSPT has limitations, notably in difficult-to-access deep pockets and molar furcations. Deep pockets (greater than 4 mm) frequently retain calculus following NSPT. To attain direct access, surgical periodontal therapy (SPT) is recommended, particularly for pockets deeper than 5 mm. Enamel matrix derivative (EMD) has emerged in recent years as a tool for periodontal regeneration when used in conjunction with NSP for infrabony defects. EMD may also have advantageous effects when combined with NSPT. The purpose of this review is to provide a thorough understanding of the effects of EMD as an adjunct to NSPT. The databases Scopus, PubMed/MEDLINE, Google Scholar, Cochrane, and Embase were systematically searched to identify relevant studies on the benefits of EMD and its use as an adjunct to NSPT. Incorporating EMD into NSPT reduces chair time, and 60% of studies demonstrated considerable benefits compared to SRP alone, according to the findings. On the basis of research, it can be concluded that EMD can be used as an adjunct to NSPT, thereby reducing the amount of time spent in the operating chair. In some cases, it can, therefore, be regarded as an alternative to surgical treatment.

## Introduction and background

The primary goal of periodontal treatment is to eliminate inflammation and restore the periodontium's integrity, which includes vital components, such as cementum, bone, and the periodontal ligament. Periodontal pocket depth (PPD), which functions as a crucial clinical parameter, frequently guides the decision-making process for treatment. Surgical periodontal therapy (SPT) and non-SPT (NSPT), the latter being a less invasive option [1,2], comprise a spectrum of periodontitis management strategies.

The initial phase of periodontitis treatment necessitates effective control of subgingival biofilm, which necessitates collaboration between dental professionals and patients. Concurrently, periodontal disease risk factors must be addressed prior to proceeding with subsequent interventions [3,4]. Various agents have been investigated as potential adjuncts to NSPT in recent scientific studies. Enamel matrix derivative (EMD), Coe-PakTM dressings, and low-level lasers are notable examples [5-7].

EMD, a composite composed of enamel matrix proteins, has been used historically in SPT to enhance periodontal regeneration, particularly in the treatment of infrabony defects [8,9]. In addition, research has revealed the beneficial impacts of EMD when incorporated into SPT [10-12].

Due to the inherent biological properties of EMD, numerous attempts have been made to investigate its potential as an adjunct to NSPT. While some studies failed to identify additional benefits from incorporating EMD into NSPT when compared to scaling and root planing (SRP) alone [13-16], others have demonstrated that EMD can indeed serve as a beneficial adjunct to NSPT, resulting in positive outcomes [11,12,17-19].

In the context of this review, we will investigate the effects of adding EMD to NSPT. This investigation will be based on randomized clinical trials that compared the effects of integrating EMD into NSPT with those of NSPT alone. The objective is to assess whether EMD can enhance the efficacy of NSPT in a scientifically rigorous and comprehensive manner.

## Review

Methodology

Cochrane, Google Scholar, PubMed MEDLINE, and Embase databases were used for bibliographic search using the following search items: (“EMD” OR “enamel matrix derivative” OR "enamel matrix proteins") and (“regeneration” OR “papilla preservation flap” OR “surgical periodontal therapy" OR “non-surgical periodontal therapy” OR “SRP” OR “deep pocket depth”). As well, a search was performed for all the reference lists of all the primary sources.

**Figure 1:**
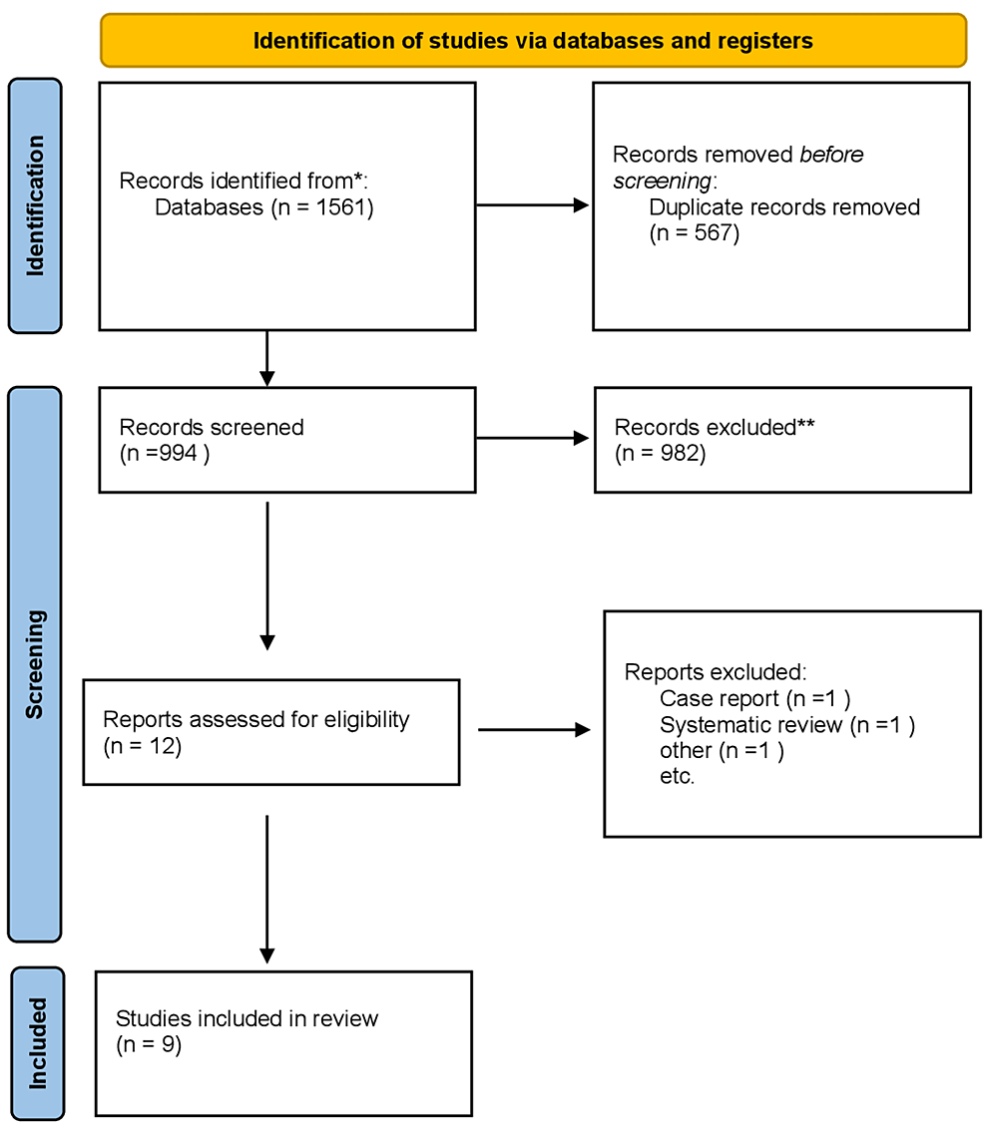
Flowchart diagram showing the inclusion and exclusion of studies

This review included all randomized clinical trials that evaluated the effect of EMD as an adjunct to NSPT in comparison to NSPT alone, which were published in the English language until September 2022. During the search process, no study based on publication date was restricted (Figure 1).

Results

According to the studies included in this review, there is a beneficial effect of using EMD as an adjunct to NSPT as it decreases chair time and improves early soft tissue healing. However, 60% of the studies showed an added benefit from using EMD as an adjunct to NSPT, whereas 40% of the studies showed no significant difference between EMD as an adjunct to NSPT compared to SRP alone.

**Figure 2:**
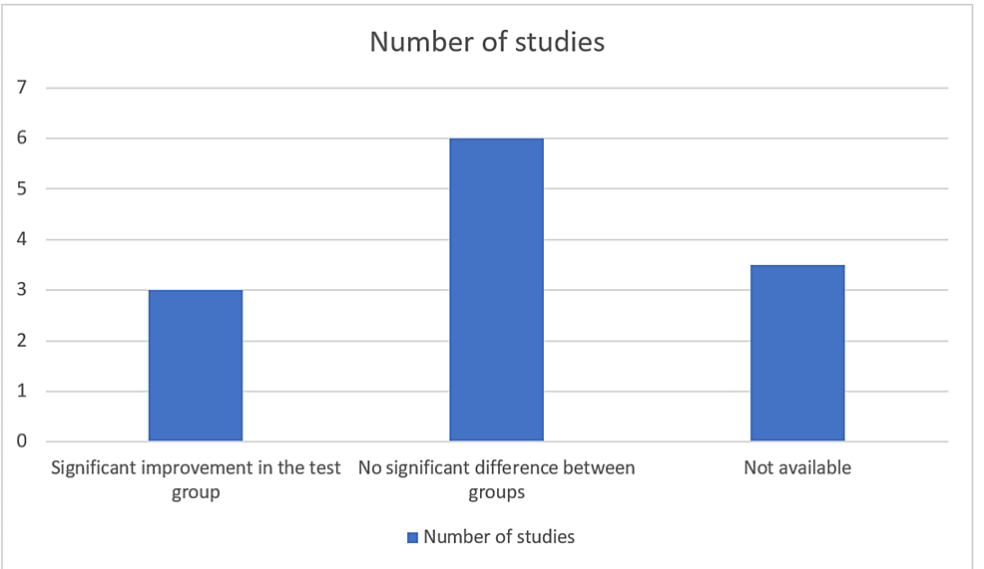
RCTs showing the effect of EMD on probing pocket depth

**Figure 3:**
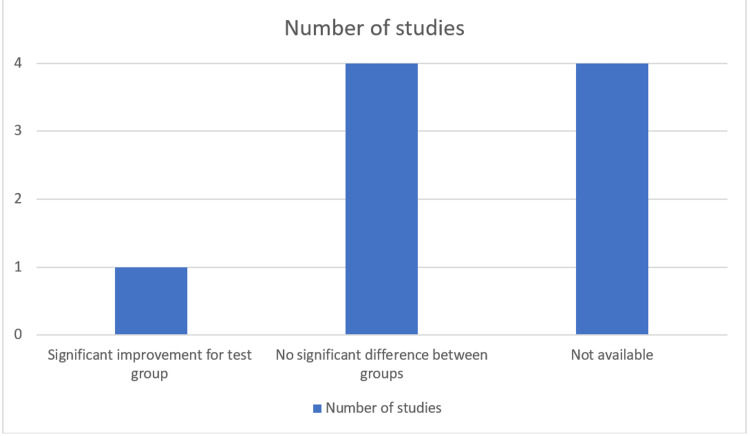
RCTs showing the effect of EMD on clinical attachment level

The lack of definitive protocol and differences in the clinical parameters measured in each study limits the ability to conclude the result. However, the number of studies with no significant difference between using EMD as an adjunct to SRP is more than studies with an added beneficial effect from using EMD, when measuring the clinical parameters PPD (Figure 2) and clinical attachment level (CAL) (Figure 3).

Discussion

Periodontitis

Periodontitis is one of the major chronic diseases worldwide [20]. Affecting the supporting apparatus of the tooth and causing irreversible damage, which is caused by the bacterial biofilm [21]. It is characterized by the imbalance between the host immune response and bacteria [22]. The risk factors for periodontitis are smoking and diabetes and other contributing factors, such as overhang restorations, anatomical factors, interproximal spaces, and occlusal trauma [23]. It may occur in adolescence or childhood, yet it is more common in patients aged more than 40 years old [23,24]. This disease can be controlled if intercepted in the early stage; nevertheless, it can cause damage and tooth loss if left untreated [25].

The main goal of periodontal treatment is the removal of the infection in order to arrest the disease, prevent its progression, and minimize symptoms. This resolution is manifested clinically by shallow PDs and the absence of bleeding on probing (BOP) [26]. However, when there are persistent deep pockets of more than 5 mm, there is a risk of disease progression, and this results in tooth loss whether there is bleeding on probing or not [27,28].

Treatment of periodontitis includes surgical and non-surgical options with behavioral changes, such as oral hygiene instructions (OHIs) and a smoking cessation program [29,30]. Daily supragingival plaque control seems to be important in order to preserve periodontal health [31,32].

According to Graziani et al. [33], it was shown that no periodontal therapy has superiority over any other treatment option [33]. Moreover, it was concluded that NSPT seeks to reduce gingival inflammation (GI) and PD and facilitates tissue healing and patient hygiene [34]. NSPT should be done in the initial cause-related therapy, and SRP should be done in deep pockets 5 mm or greater using manual or ultrasonic scalers [23], while SPT provides accessibility for SRP, especially in sites with furcation and infrabony defect involvement, and provides bone and gingival contouring for facilitating self-oral hygiene [35,36]. Residual calculus accumulation was found in pockets more than 4 mm after SRP [37]; thus, SPT should be done after the hygienic phase in the remaining deep pockets (more than 5 mm) in order to avoid mechanical damage of the periodontium. In addition, surgical therapy should be delayed if there is no adequate biofilm removal [33,38-40].

Increased probing depth following treatment means that there is an infrabony defect (angular defects); thus, this angular defect seems to worsen the prognosis of the teeth [41].

Despite the fact that surgery and NSPT result in improvement of the clinical outcome, it is characterized by long junctional epithelium, which means repair and not regeneration (formation of new cementum, periodontal ligament (PDL) fibers, and new alveolar bone) [42]. Moreover, regeneration of periodontal tissue is the goal of periodontal treatment [43,44], and different surgical techniques include implantation of bone grafts or/and substitutes in order to achieve periodontal regeneration [2,45,46].

Some systematic reviews showed that these regenerative techniques result in better clinical outcomes in terms of clinical attachment level (CAL), PD, and hard tissue fill compared to open flap debridement (OFD) alone [2]. Meanwhile, other studies showed that these clinical outcomes can be maintained long-term in cases where plaque control is maintained [9,45,47-50].

EMD

In 1997, EMD was introduced [51], and it is produced from the enamel of a developing porcine tooth. Consisting of enamel matrix proteins (EMPs), amelogenin is the most important protein and propylene glycol alginate (PGA) [52]. PGA has antibacterial properties [5]. Cells of periodontal ligaments are favored by EMD over epithelial and gingival cells, which do not consist of cytotoxic but cytostasis (inhibition of cells), having the least effect on epithelial cells [9]. Moreover, EMD did not show any side effects, such as allergic or incompatibility [53,54].

EMPs are secreted from Hertwig’s epithelial root sheet, which is able to promote periodontal regeneration [55]. It was shown that it induces bone cells (osteoblasts) and PDL cells and stimulates the proliferation of osteogenesis [35,56-59], and it was also shown that it stimulates the growth of new blood vessels [60,61].

EMD has been used in regenerative periodontal therapy; in some studies, it has been shown that there are better clinical outcomes using EMD in SPT compared to OFD alone [62].

A randomized clinical study by Heijl et al. showed that the CAL, when using EMD and surgery (test group), is 2.2 mm, while it is 1.7 mm when applying OFD alone (control group). The radiographs in the test group showed that there is a 2.6 mm bone fill, and 66% of the defect is filled, while the control group did not show any bone fill [63]. Furthermore, the study by Froum et al. [64] showed three times bone fill in the test group compared to the control group, and 74% fill of the defect filled versus 23% fill of the defect in the control group.

Some studies included that the application of EMD in conjunction with surgical treatment caused regeneration of new PDL, cementum, and bone in angular bone and class II furcation defects [9,52,65], Moreover, it was concluded that EMD improves soft tissue healing in the SPT in the post-operative period [66].

Application of the EMD in SPT

The key elements for successful regeneration are space provision and clot stabilization in infrabony defects [67-69]. Although EMD has limited space-making potential due to its gel-like consistency, this will not provide sufficient soft tissue support [9,67]. Thus, root instrumentation should be done after flap elevation using a simplified papilla preservation flap (SPPT) or modified papilla preservation flap (MPPT) in order to obtain primary closure and clot stabilization, thus obtaining better clinical results [70,71].

Ethylenediaminetetraacetic acid (EDTA) is always used before the application of EMD as a root surface bio modifier. It has a natural PH; thus, the vitality of the tissue is preserved [72]. It was shown that collagen fibers are exposed, the smear layer is removed, and dentinal tubules are demineralized by EDTA [73]. Thus, the root is conditioned with EDTA 24% for two minutes, followed by the application of EMD [74].

EMD as an Adjunct to NSPT

In order to preserve the soft tissue and improve wound healing, a minimally invasive technique is preferred over surgical therapy [75]. EMD showed a beneficial effect when used in SPT. Thus, this effect may have benefits when using EMD with NSPT.

Some clinical studies evaluated the effect of the EMD as an adjunct to NSPT but failed to find additional benefits when compared to NSPT alone [13,14].

Other randomized clinical studies showed that there are no additional benefits from using EMD with NSPT when compared to SRP alone in terms of CAL and PD, but it was revealed that using EMD with NSPT has less postoperative pain [16]. While in another randomized clinical trial, it was revealed that, in the test group, two of 10 teeth showed insignificant new cementum and new bone formation; however, it failed to show a significant benefit from using EMD as an adjunct to NSPT [52]. Furthermore, a significant improvement after 12 months of treating infrabony defects, less than 8 mm, was observed with minimally invasive non-surgical techniques, but failed to show significant improvement in radiographic outcomes when using additional EMD [5]. By incorporating both non-surgical and minimally invasive surgical perspectives, the systematic review is better equipped to meet the diverse requirements of patients and clinicians. This strategy is consistent with the dynamic nature of periodontal therapy and the recognition that there is no single modality that is universally superior. This study contributes a more holistic comprehension of how EMD can enhance various periodontal treatment strategies and thus fits well within the existing literature.

In a case report of four cases, three cases had new cementum and new bone formation; thus, it was found, in the case report, that EMD has a beneficial effect and can be used after NSPT in deep pockets. According to the study, it was concluded that EMD as an adjunct to NSPT is used in patients who refuse surgical treatment [18]. In addition to this, it was concluded in a randomized clinical study that there is no change in the clinical parameters when comparing EMD with SRP versus SRP alone (Table 1). But using EMD inhibits the development of Prevotella and Porphyromonas gingivalis [17].

**Table 1:** Summary of the randomized clinical trial (RCTs) comparing EMD as an adjunct to non-surgical periodontal therapy versus non-surgical periodontal alone

Authors, year, and study design	Age	Sample size	Test group	Control group	Follow-up	Clinical and histological parameters	Clinical evidence	
Anoixiadou et al. [5], 2022, RCT	More than 18 years	36 patients with infrabony defects	18 patients, minimally invasive surgical technique (MINST)+ EMD	18 patients, MINST alone	6 and 12 months	CAL, BOP, and PPD	EMD has no added benefit over MINST alone. EMD significantly decreases PPD with BOP in sites with a baseline pocket depth of 8 mm or less (92% in the test group and 69% in the control group).	
Schallhorn et al. [12], 2021, Split mouth RCT	18-85 years old	51 patients with moderate to severe periodontitis	SRP+EMD, second application 2-3 weeks later	SRP alone	12 months	CAL, PPD, and BOP	A significant improvement for both groups in PPD and CAL. A significant improvement in BOP, CAL, and PPD in test sites compared to control sites. Sites that are converted to sites that no longer needs treatment: 65% for control sites and 79% for test sites.	
Gutierrez et al. [13], 2003, Split mouth RCT	More than 18 years old	22 patients	22 sites with pocket depth of 5 mm or greater and angular bone defect, with more than 3 mm. EMD application with SRP	22 sites SRP without additional treatment	3 months	CAL, PPD, PI, and bleeding index (BI)		No significant difference in all clinical parameters between two groups. Mean PPD 1.47±0.3 mm for test sites, and 1.87±0.4 mm for control sites.
Mombelli et al. [14], 2005, Split mouth RCT	25–65 years old	16 patients with moderate to advanced periodontitis	16 sites EMD +SRP	16 sites, SRP alone	2, 6, and 12 months	GI, PI, BOP, and PPD		No added benefit from EMD as an adjunct to non-surgical periodontal therapy.
Sculean et al. [15], 2003, RCT	NA.	16 patients with one intrabony defect	1. SRP (hand)+EMD, 2. SRP (ultrasonic)+EMD	SRP alone	6 months	Histological evaluation, CAL, and PPD		PPD reduction and CAL gain in all groups, but no true connective tissue attachment in all three modalities.
Wennström et al. [16], 2002, Split mouth RCT	NA	84 patients with moderate to severe periodontitis	84 sites, EMD +SRP	28 sites, SRP alone	1, 2, and 3 weeks	PPD, BOP, GI, post-treatment discomfort		EMD enhanced early soft tissue healing (at 1 week proportion of people having minimal discomfort were 54% for test group and 31% for control group, but no significant difference at a three-week follow-up. No significant difference in the clinical parameters between groups at a three-week follow-up.

According to Schallhorn et al., different results in the studies are caused by a lack of standardized protocol. The application of EMD for a second time on the root during healing will prolong the presence of EMD on the root surface, and this decrease the potential of washing out the material. This double-blinded randomized clinical study concluded that EMD can be used as an adjunctive to non-surgical periodontal treatment in implant-associated diseases. Furthermore, a randomized clinical trial of 51 patients found that the control and the test groups have significant improvement in the clinical attachment level and PPD. Nonetheless, the use of the EMD improves bleeding on probing and increases the number of healthy pockets (less than 5 mm); these pockets were converted to sites that no longer need surgery, a standardized workflow in this study [12].

In summary, the study demonstrated that a flapless technique employing EMD in infrabony defects reduces chair time compared to the minimally invasive technique (MIST) [11]. Additionally, when EMD is used as an adjunct to NSPT for peri-implant mucositis, it provides some additional advantages in terms of PPD and CAL, but it may not result in a full recovery, and its efficacy compared to NSPT alone may not be particularly significant [19]. Only one study investigated the effectiveness of EMD application as an adjunct to non-surgical retreatment therapy after three months after the initial NSPT and concluded that the application of EMD in the re-instrumentation session increases significantly the frequencies of pocket closure, with no bleeding on probing compared to re-instrumentation alone (Table 1) [76].

Limitations

The lack of a definitive protocol for the EMD application between the studies, variability in follow-ups, and the parameters measured will limit the review from having a definitive conclusion. Moreover, due to heterogenicity in lifestyle in each study because of different countries, more evidence is required to confirm the beneficial effect of EMD as an adjunct to NSPT.

## Conclusions

While some studies did not observe additional benefits from using EMD as an adjunct to NSPT compared to non-surgical therapy alone, this may be attributable to the techniques used. These investigations may have employed EMD in a manner that increased the possibility of material washout by the blood. However, when EMD is utilized in a less invasive manner during NSPT, it can substantially reduce chair time and provide greater patient comfort than SPT. Consequently, EMD can still be considered as an adjunct to NSPT, particularly in deep pockets (greater than 5 mm), where surgical alternatives are unavailable or refused by the patient.

In conclusion, EMD can be a valuable instrument when combined with NSPT. It reduces chair time and provides a more comfortable experience for patients, especially those who are ineligible for or refuse surgical treatment.
